# Engineering innovations in medicine and biology: Revolutionizing patient care through mechanical solutions

**DOI:** 10.1016/j.heliyon.2024.e26154

**Published:** 2024-02-15

**Authors:** Eddie Gazo Hanna, Khaled Younes, Rabih Roufayel, Mickael Khazaal, Ziad Fajloun

**Affiliations:** a College of Engineering and Technology, American University of the Middle East, Egaila, 54200, Kuwait; b École Supérieure des Techniques Aéronautiques et de Construction Automobile, ISAE-ESTACA, France; c Faculty of Sciences 3, Department of Biology, Lebanese University, Campus Michel Slayman Ras Maska, 1352, Tripoli, Lebanon; d Laboratory of Applied Biotechnology (LBA3B), Azm Center for Research in Biotechnology and Its Applications, EDST, Lebanese University, 1300, Tripoli, Lebanon

**Keywords:** Biology, Biomechanics, Biomedical engineering, CFD, Composites, Medicine, Nanomechanics

## Abstract

The overlap between mechanical engineering and medicine is expanding more and more over the years. Engineers are now using their expertise to design and create functional biomaterials and are continually collaborating with physicians to improve patient health. In this review, we explore the state of scientific knowledge in the areas of biomaterials, biomechanics, nanomechanics, and computational fluid dynamics (CFD) in relation to the pharmaceutical and medical industry. Focusing on current research and breakthroughs, we provide an overview of how these fields are being used to create new technologies for medical treatments of human patients. Barriers and constraints in these fields, as well as ways to overcome them, are also described in this review. Finally, the potential for future advances in biomaterials to fundamentally change the current approach to medicine and biology is also discussed.

## 1 Introduction

Mechanical engineering is a broad discipline that develops from the need to design and construct anything from small individual components and devices, extending from microscale sensors to enormous systems like spacecraft and machine tools [1]. To understand mechanical systems, mechanical engineers study materials, solid and fluid mechanics, thermodynamics, heat transport, control, instrumentation, design, and manufacturing [2]. They are tasked with bringing a product from conception to market. The human body is a very complex system, and as mechanical engineering is concerned with bodies in motion, it is also a part of this discipline [2].

Nowadays, medical science and biological sciences both embrace mechanical ideas and theories. Examples include the fundamental role of mechanics in immunology, orthopedics, and the complete reliance on mass transport and diffusivity equations for the understanding of cardiovascular physiology and pathology [3]. Currently, the combination of mechanical engineering with biology and medicine contributes in the discovery of new treatments for serious conditions and spurs a great deal more advancements in the biomedical sector. Since, some topics relating mechanical engineering to biology and medicine are detailed in Table 1.

**Table 1:** Topics relating Mechanical Engineering to Medicine and Biology [4].

Topics Relating Mechanical Engineering to Medicine and Biology	
Biomechanics	Biomaterials
Gait Analysis	Metals
Joint Mechanics	Ceramics
Muscle Mechanics	Polymers
Spine Mechanics	Composites
Soft Tissue Mechanics	Hydrogels
Tissue Engineering Mechanics	Tissue Scaffolds
Sports Biomechanics	Surface Modification
Rehabilitation Biomechanics	Biocompatibility
Biomechanics of Injury	Biodegradability
Biomechanics of Prosthetics	Drug Delivery
**Bioinstrumentation**	**Biomedical Imaging**
Biosensors	Magnetic Resonance Imaging (MRI)
Diagnostic Tools	Computed Tomography (CT)
Monitoring Devices	Ultrasound Imaging
Wearable Sensors	X-ray Imaging
Imaging Equipment	Optical Imaging
Point-of-Care Testing	Molecular Imaging
Microfluidic Devices	Positron Emission Tomography (PET)
Lab-on-a-Chip	Single-Photon Emission Computed Tomography (SPECT)
Robotics	Photoacoustic Imaging
Non-invasive Techniques	Multimodal Imaging
**Bionanotechnology**	**Biosensors**
Nanoparticles	Optical Biosensors
Nanofibers	Electrochemical Biosensors
Nanocomposites	Piezoelectric Biosensors
Drug Delivery	Acoustic Wave Biosensors
Biosensors	BioMEMS Biosensors
Tissue Engineering	Aptamer-Based Biosensors
Molecular Imaging	Nano-Based Biosensors
Nanorobotics	Wearable Biosensors
Nanotoxicology	Implantable Biosensors Point-of-Care Biosensors
**Cardiovascular Mechanics**	**Computational Biology**
Blood Flow	Computational Modeling
Heart Mechanics	Data Analysis
Artery Mechanics	Simulation
Atherosclerosis	Optimization
Heart Valve Mechanics	Machine Learning
Hemodynamics	Artificial Intelligence
Cardiovascular Devices	High-Performance Computing
Medical Imaging	Genomics
Cardiovascular Modeling	Proteomics Systems Biology
**Cell Mechanics**	**Drug Delivery**
Cytoskeleton Mechanics	Targeted Drug Delivery
Cell Adhesion	Controlled Release
Cell Migration	Nanoparticle Delivery
Cell Signaling	Biodegradable Delivery
Cell Stiffness	Implantable Devices
Cell Differentiation	Oral Drug Delivery
Cell Mechanics Modeling	Transdermal Drug Delivery
Cell-Material Interactions	Inhalation Drug Delivery
Mechanobiology	Injectable Drug Delivery
**Medical Devices**	
Implantable Devices	
Diagnostic Devices	
Monitoring Devices	
Therapeutic Devices	
Prosthetics	
Assistive Devices	
Rehabilitation Devices	
Surgical Tools	
Smart Devices	

This review explores the intersection of mechanical engineering with medicine and biology, delving into specific topics that have reshaped these fields. We will navigate through key subjects, such as.

1. **Application of Biomaterials in the Medical Field:** Exploring the role of advanced materials in medical applications and specifically focusing on the use of polymers and composites in the medical field.
2. **Biomechanics Applications:** Showing recent type of implants, the way they are used, there biocompatibility and mechanical evaluation.
3. **Nanomechanical Materials in Medicine:** Analyzing the utilization of nanomechanics to unlock new possibilities in medicine and specifically focusing on carbon nanotubes applications in this field.
4. **Numerical Simulation Tools for Medical Applications:** Examining the role of numerical simulation tools in solving complex medical challenges, including finite element simulation and computational fluid dynamics applied in the human cardiovascular and respiratory systems.
2. Application of Biomaterials in medical field

Biomaterials are materials that are biocompatible and can be used in medical applications such as implants, drug delivery, and tissue engineering. They have revolutionized the medical field, improved patient outcomes and enabling new treatments. Table 2 shows different types of biomaterials, their properties and applications. Subsequently, we will focus on the use of polymers and composites in the medical field, which have gained significant attention due to their versatility, biocompatibility, and mechanical properties that can be tailored to specific medical requirements.

**Table 2:** Different types of functional biomaterials, their properties and applications in different fields.

Biomaterials	Applications	Properties
1. Metals (e.g., titanium, stainless steel, Stainless steel) 2. Titanium alloy 3. Cobalt-chrome alloys 4. Gold 5. Silver 6. Platinum 7. Hydroxyapatite (HA) 8. Tricalcium phosphate (TCP) 9. Biphasic calcium phosphate (BCP)	1. Implants (e.g., joint replacements, dental implants, surgical instruments, Cardiac pacemaker, Hip implant and Knee implant) 2. Joint replacement, surface coatings on total joint replacements, cellular scaffolds 3. Joint replacement, bone fracture fixation 4. Fillings and crowns, dental electrodes 5. Pacemaker wires, suture material, dental amalgams 6. Electrodes for peripheral nerve stimulation	High strength, durability, biocompatibility, corrosion resistance
Ceramics (e.g., alumina, zirconia)	Bone grafts, dental implants, joint replacements	High strength, biocompatibility, wear resistance, brittle
Polymers (e.g., polyethylene, polyurethane, polylactic acid, polylactic-*co*-glycolic acid and polyhydroxyalkanoates)	Contact lenses, wound dressings, drug delivery, tissue engineering, bone regeneration	Low density, flexibility, biocompatibility, degradation over time
Composites (e.g., carbon fiber reinforced polymers)	Bone implants, dental implants, prosthetics	High strength, stiffness, biocompatibility, manufacturing challenges
Biomimetic materials (e.g., hydrogels)	Tissue engineering, drug delivery, wound healing	Biocompatibility, water absorption, biodegradability, low mechanical strength

In recent decades, the medical field has witnessed a surge in the application of composites, polymers and other biomaterials, especially in tissue engineering and other therapeutic areas. These advancements have been fueled by the integration of various innovative technologies, making treatments more effective and versatile.

Composites are materials that combine two or more constituents. Combining polymers with other materials can improve the properties of these individual constituents, such as their mechanical strength, surface characteristics, and biocompatibility [[5], [6], [7]]. However, there are limitations associated with composites in the biomedical sector. Ensuring the biocompatibility and long-term performance of these materials remains a paramount concern. Some composites might not always exhibit long-term stability within the body and could elicit immune responses or show reduced biocompatibility over time [8]. Surface modifications and functionalization techniques, including surface coatings and the incorporation of bioactive molecules, offer promising avenues to enhance the performance and functionality of these composites. This not only ensures their long-term performance but also fosters enhanced cell-material interactions, optimizing therapeutic efficacy.

When selecting polymers for biomedical applications, biodegradability and stability are paramount. One lingering challenge is ensuring these degrading polymers refrain from releasing toxic by-products detrimental to the body [9]. Homo and copolymers like polyamides, polyesters, and others are known for their hydrolytic degradability [10]. Often termed smart and biopolymers, their predominant application is in medicine and biotechnology. The integration of these polymers with advanced technologies like 3D printing and bioprinting can revolutionize their utility, allowing precise fabrication of intricate structures, tailored release systems, and patient-specific implants, ushering a new era of personalized medicine [11].

Polylactic Acid (PLA) is one such polymer renowned for its biodegradability and biocompatibility. Its distinct properties, such as regulated degradation rates, mechanical strength, and processability, have positioned it as a preferred choice for tissue engineering scaffolds, drug delivery systems, and implantable devices. Studies on PLA-based systems have shown encouraging results in bone regeneration, wound healing, and controlled drug release [[12], [13], [14]].

Another notable polymer is Polylactic-*co*-glycolic acid (PLGA), a copolymer derived from lactic acid and glycolic acid. PLGA amalgamates the beneficial properties of its constituents, boasting adjustable degradation rates, mechanical robustness, and biocompatibility. Owing to its versatility, PLGA has been extensively researched for applications in drug delivery, tissue engineering, and wound healing, with particular emphasis on its microspheres, nanoparticles, and scaffolds for controlled drug release and tissue regeneration [[15], [16], [17]].

Further, Polyhydroxyalkanoates (PHA) are biodegradable polymers produced by microorganisms. Their exceptional biocompatibility and biodegradability make them ideal for tissue engineering, wound healing, and drug delivery applications. In practice, PHA-based scaffolds and implants have demonstrated potential in promoting tissue regeneration and controlled drug delivery [18].

On the other hand, polymeric nanoparticles are of particular interest in the realm of cancer therapy. They have been utilized as drug carriers for targeted drug delivery, ensuring that the therapeutic agents are delivered specifically to tumor sites while minimizing systemic side effects [19]. For instance, polymeric micelles, which are self-assembling nanoparticles, have been shown to increase the solubility of hydrophobic anti-cancer drugs, enhancing their bioavailability and therapeutic efficacy [20].

The development of polymer-drug conjugates further underscores the importance of polymers in oncology. By directly attaching the drug molecule to the polymer, researchers have been able to capitalize on controlled drug release mechanisms. This ensures a sustained release of the therapeutic agent over an extended period, reducing the frequency of dosing and potentially mitigating adverse reactions [21].

Furthermore, polymers have been employed in creating hydrogels and implantable devices for localized drug delivery. These polymeric systems can be implanted directly into the tumor site, offering a continuous and localized release of chemotherapeutic agents. Such an approach not only enhances drug bioavailability but also significantly reduces the systemic toxicity often associated with traditional chemotherapy treatments [22].

Polymers also play a significant role in the burgeoning field of photothermal therapy (PTT) for cancer. In PTT, polymeric nanoparticles are designed to absorb near-infrared light, leading to localized heat generation. This heat can destroy cancer cells directly, providing a non-invasive method of treating tumors. The integration of polymers ensures that these nanoparticles remain stable and can be directed specifically to tumor sites, maximizing the therapeutic potential of PTT [23].

Beyond treatment modalities, polymers have also been incorporated into diagnostic platforms for cancer. Polymer-based nanoprobes, often combined with imaging agents, have been designed to target specific cancer biomarkers. These can be utilized in conjunction with imaging modalities like MRI or PET scans, offering clinicians a detailed, molecular-level view of the tumor, aiding in both diagnosis and treatment planning [24].

## 2 Biomechanics applications

### 2.1 Hydrocephalus shunts

**Fig. 1:**
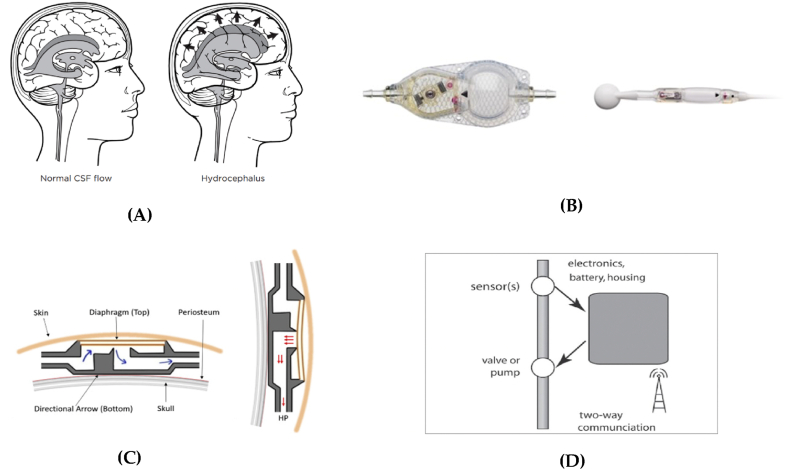
Hydrocephalus shunts. (A) human brain without and with hydrocephalus [22]. (B) on the left: CERTAS™ Plus Programmable Valve designed by CODMAN and on the right: HAKIM Programmable Valve designed by CODMAN [28,29]. (C) schematic illustration of the Anti-Siphon device; on the left: in horizontal and on the right: in vertical positions. (D) conceptual framework for a smart shunt.

Hydrocephalus is a condition in which there is an abnormal accumulation of cerebrospinal fluid (CSF) within the ventricular system of the brain. This can cause an enlargement of the ventricles and a corresponding increase in intracranial pressure, which can lead to a variety of symptoms such as headaches, nausea, vomiting, and cognitive impairments [25,26] (Fig. 1, A). One way to treat hydrocephalus is to surgically insert a shunt, which is a tube that is inserted into one of the ventricles and then routed to another part of the body, such as the peritoneal cavity, where the excess cerebrospinal fluid can be absorbed. The shunt is typically controlled by a valve that is located on the side of the head and can be adjusted to regulate the flow of CSF [27].

Engineering designs of shunts have undergone significant advancements over the years, with the primary focus being on making the devices more reliable, effective, and easier to use. Mechanical engineers are often involved in the design, development and testing of shunt systems to ensure they function properly and are safe for use in patients. This can include evaluating the biomaterials used for the different components of the shunt and optimizing the design for ease of use and durability. One of their key challenges is ensuring that the devices remain functional for long periods of time without the need for revision surgery [[30], [31], [32]]. Examples of shunt designs created by engineers include i) programmable shunts which have an adjustable pressure valve that can be remotely adjusted by a healthcare provider, allowing for more precise control of fluid drainage [[28], [29], [33], [34], [35]] (Fig. 1, B); ii) anti-siphon shunts which are designed to prevent fluid from flowing back into the brain, a condition known as “siphoning” [36,37]. This device made by Natus Medical in Pleasanton, operates as follows: when it is in a horizontal position, the diaphragm is moved away from the crown seat as long as the pressure inside the skull is greater than the pressure outside (which is slightly higher than normal atmospheric pressure). However, when the device is in a vertical position, the negative pressure created by the fluid attracts the diaphragm towards the crown seat, stopping any flow of cerebrospinal fluid (Fig. 1, C) [38]; iii) smart shunts with built-in sensors which have built-in sensors that monitor the flow of fluid and alert healthcare providers if there are any issues with the shunt function [[39], [40], [41]]. A concept of an intelligent smart shunt related to an implantable system made up of hardware and algorithms aimed at regulating cerebrospinal fluid (CSF) drainage is shown in (Fig. 1, D). The function of this shunt is based on feedback from various measurements. The majority of smart shunts have a similar structure and components, including: sensors such as intracranial pressure (ICP) and CSF flow rate; a fluid control mechanism like a pump or valve; an actuator to operate the pump or valve; an electrical component isolating housing; a battery that may be rechargeable; and communication capabilities for adjusting device settings and accessing sensor data [42,43].

Finally, shunt surgery is usually considered to be a safe procedure, but there can be complications such as infection, blockage, or malfunction of the shunt. It is important for patients who have had a shunt placed to be followed closely by a neurologist or neurosurgeon to ensure that the shunt is functioning properly and to address any issues that may arise [27].

### 2.2 Implants

In this section, we introduce some of the most straightforward mechanical implants used in medicine by surgeons to treat deformity, stabilize and strengthen, and facilitate fusion of bone structures across the body such as implants for extremities, spinal, trauma, CMF and ortho-implants.

#### 2.2.1 Extremities implants

Extremity implants are medical devices that are used to replace or support bones, joints, and other structures in the extremities, such as the arms and legs. They are designed to restore function and alleviate pain for individuals with conditions such as arthritis, injury, or congenital defects. According to recent studies [[44], [45], [46], [47]], the use of extremity implants has increased significantly in recent years due to advancements in biomaterials and design. These implants are made from biocompatible materials such as titanium and ceramic, which have proven to be safe and durable over time.

Joint replacement, such as a knee or hip replacement, is an example of an extremity implant. These prosthetic joints replace damaged ones, restoring mobility. Recent studies indicate that joint replacement surgeries are among the most successful in orthopedic surgery, offering high levels of patient satisfaction and improved quality of life [[48], [49], [50]]. Mechanical stability in these implants is crucial as they must bear the body's full weight, resist various forces, and allow smooth movement. The materials, primarily cobalt-chromium alloys, stainless steel, and biocompatible polymers, are chosen for long-term load-bearing. These implants mimic the knee's natural biomechanics, potentially prolonging their lifespan. Design variations include fixed-bearing and mobile-bearing implants, with the latter providing smoother articulation by letting the polyethylene insert move with the tibia or femur [51]. Yet, wear of some components can affect long-term performance [52], and incorrect surgical placement might lead to undue stresses, causing potential implant failures [53].

For joint replacements, some surgeons prefer modular implant components because they can more precisely customize the implant to the patient's individual anatomy. For instance, it can be crucial to correct the femoral neck angle and take leg length disparities into account while performing hip replacements. These components use various metals for a variety of functions that improve bone attachment and minimize friction [[54], [55], [56], [57], [58], [59]]. However, utilizing different alloys might present difficulties, such as fretting from micro-movements and probable galvanic corrosion, which can result in metal debris and ion migration [60].

**Fig. 2:**
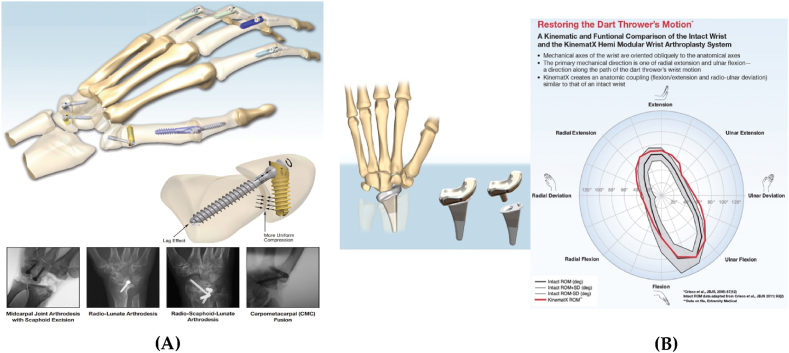
Extremities implants. (A) UpperFiX Fusion Fixation [48]. (B) Restoring the Dart Thrower's motion [49].

On the other hand, extremity implants can also include external devices such as braces and splints, which are used to provide support and protection to injured or weakened extremities. The use of these devices has been shown to be effective in treating conditions such as wrist fractures and sprains, and can help to improve the function and stability of the affected extremity [61,62]. Other examples of applications using extremity implants include ankle alignment systems such as the Align X Ankle Fusion System made by Extremity medical [63]. With the use of this implant, the surgeon is able to perform arthrodesis, a surgical procedure that artificially induces joint ossification between two bones, by applying rigidity and compression across the ankle joint [63]. Another good example of extremities implants, also made by Extremity Medical is the Upper FIX designed for fusion of hand articulations (Fig. 2, A). With fixed angles of his choosing (25, 30, 45, 60 and 75°), the surgeon applies strong uniform compression to the joints again to perform arthrodesis in this case [64].

Extremities implants can be found in more complex and more permanent forms. They can play a role in preserving articulation and restoring the complete range of motion for the patient. KinematX (made by Extremity Medical) is a modular implant that adapts to the patient's anatomy and restores the “Dart Thrower's Motion” in the wrist [65] (Fig. 2, B).

This type of implants has significantly improved the quality of life for individuals. However, these implants may have certain side effects that can affect a patient's overall health and well-being. Complications such as infection, nerve damage, and implant failure can occur and require additional surgery or medical attention. Additionally, the psychological impact of living with an extremity implant can also be significant, including issues such as body image concerns, depression, and anxiety. Research studies have identified these potential side effects, and ongoing efforts are being made to minimize their occurrence and improve the outcomes for patients with extremity implants [66,67].

#### 2.2.2 Spinal implants

Spinal implants, essential in the treatment of spinal pathologies, are designed to provide structural support to the spinal column, manage pain, and improve mobility in patients with debilitating spinal conditions [[68], [69], [70]]. Predominantly crafted from materials like titanium and its alloys, cobalt-chromium alloys, and stainless steel, the yield strength for the commonly utilized titanium alloy (Ti–6Al–4V) is approximately 830–900 MPa, with an elastic modulus ranging from 110 to 117 GPa [71]. The range of designs includes pedicle screws, interbody cages, dynamic stabilization devices, and artificial discs.

Pedicle screws provide robust fixation, especially when combined with rods for segmental stabilization, aiding in the stabilization of the spinal column, pain reduction, and function improvement. Interbody cages, primarily used in fusion surgeries, have seen various designs that are tailored to the anatomical and biomechanical needs of specific spinal regions [72]. Dynamic stabilization devices are designed to maintain motion while providing stability, commonly used to treat spondylolisthesis and spinal instability [73,74]. In contrast, artificial discs, replacing natural discs, mimic their function allowing spinal motion while ensuring stability [75,76].

One notable spinal care solution is provided by DePuySynthes from Johnson-Johnson [77], with one of their prominent implants being the DePuy Synthes Spine SYMPHONY™ Occipito-Cervico-Thoracic (OCT) System [78]. This system offers an assortment of instruments and implants, addressing the needs of fixation and alignment in the posterior upper spine area.

These implants offer a harmonized balance between rigidity and flexibility, encouraging osseointegration and reducing complications. However, despite their efficacy in managing spinal conditions and improving quality of life, complications like infection, nerve damage, implant migration, and bone loss can emerge. Such complications may necessitate additional surgical interventions, potentially leading to chronic pain, limited movement, and reduced activity levels, impacting the overall health and well-being of patients [79,80].

#### 2.2.3 Craniomaxillofacial implants

Craniomaxillofacial implants (CMF) are medical devices used to treat a variety of cranial and facial conditions, including jaw and cranial fractures, congenital deformities, and facial trauma [81,82]. They are designed to restore the normal anatomy and function of the cranial and facial bones, improving the appearance and quality of life of patients [83]. CMF implants come in a variety of designs, including plates and screws, jaw implants, and cranial implants [84]. The choice of implant design depends on the specific needs of the patient and the nature of the cranial or facial condition being treated.

DePuySynthes offers personalized solutions with advanced technology allowing facial reconstruction, orthognathic surgery, trauma, distraction and cranial reconstruction [85]. Personalized solutions also mean that virtual surgical planning is used to ensure accuracy and efficiency of the treatment. Since not all surgeons have the appropriate software skills to prepare such procedures, clinical engineers with mechanical engineering training are required to help plan the intervention and select the best course of action. Once more, it is a system of screws and a combination of plates made to fit the patient's anatomy that enable the treatments seen in the figures above [85]. Another type of CMF implant is a jaw implant, which is used to correct jaw misalignments or jawbone deficiencies. Studies showed that jaw implants have been successful in restoring the functional and aesthetic aspects of the jaw, and can provide long-lasting results [86]. Plates and screws are commonly used to treat jaw and cranial fractures, as well as facial trauma. They are designed to stabilize the affected bone, reducing pain and restoring normal anatomy [87]. Cranial plates and screws are made of biocompatible materials, such as titanium, and can be implanted through minimally invasive surgical techniques [87]. A third type of CMF implant is a cleft lip and palate implant, which is used to treat patients with congenital defects that affect the upper lip and palate. Recent studies reported that cleft lip and palate implants have been shown to be effective in restoring the normal anatomy and function of the lip and palate, and can greatly improve the patient's quality of life [88,89].

While these implants have greatly improved the quality of life for many patients, they may also cause certain side effects. Patients with craniomaxillofacial implants may experience facial asymmetry, reduced facial sensation, and difficulty speaking or chewing, which can have a significant impact on their overall health and well-being [90,91].

#### 2.2.4 Ortho trauma implants

An orthopedic trauma is a physical injury that involves fractures, subluxation or dislocation of bones and joints [92,93]. The decision to replace a joint, bone, or cartilage with an implant due to damage or deformity is not an easy one. Surgery is never without risk [94], but occasionally the advantages outweigh the risks, as in the case of patients who might fear being bound to a wheelchair when regaining the ability to walk is entirely feasible [95]. In order to restore the physiology or anatomy of the damaged elements following such traumas, the surgeon may use an implant or a system of implants. As previously mentioned, such intervention may be either temporary or permanent. The doctor choses the implant based on weight, size and type most adapted to the patient. Although increasingly innovative materials like ceramics and polymers are being employed, the majority of the time the implants are either made of titanium or stainless steel wrapped in a plastic coating that functions as artificial cartilage. These metals are known for their incredible strength and toughness and they can persist for more than ten years before needing to be replaced [95].

#### 2.2.5 Mechanical evaluation of implants

When selecting metals for various applications, it's crucial to consider their physical attributes, such as density, melting point, specific heat, and other thermal properties. The density of a metal, in particular, plays a pivotal role in determining its strength and stiffness relative to its weight.

Corrosion resistance is a primary concern, especially influenced by the metal's composition and its exposure to environmental factors. Adopting the right metal for specific corrosive conditions becomes an effective mitigation strategy. Notably, metals like nonferrous, stainless steel, and some non-metallic substances are inherently resistant to corrosion due to protective layers they form. Titanium, for example, forms a protective titanium oxide layer, TiO_2_, while stainless steels benefit from a chromium oxide layer. If these protective layers are compromised, they typically regenerate, shielding the metal anew.

Beyond the physical, metals possess mechanical properties that determine their response to external stresses. The tension test, often adhering to ASTM standards, is a conventional method for evaluating attributes like strength, ductility, and toughness. Furthermore, hardness, a key property, measures a metal's resilience against specific deformations, with several techniques established for its assessment.

Table 3 shows mechanical properties of some alloys used for implant. The composition of these alloys plays a significant role in determining their mechanical properties. For instance, adding Al and V to pure titanium markedly improves its tensile resilience. The treatment processes, such as annealing, further influence these properties.

**Table 3:** Example of metals used for implants and their mechanical properties. *Under annealed condition except for WE_43_ which was solution heat-treated and artificially aged (T6). YS = yield strength, UTS = ultimate tensile strength, YM = Young's modulus. Extracted from Hermawan et al. [96].

Metals	Main alloying composition (wt%)	Mechanical Properties			
		YS (MPa)	UTS (MPa)	YM (GPa)	Max elongation (%)
Stainless steel: 316L type (ASTM, 2003)	Fe; 16-18.5Cr; 10-14Ni; 2-3Mo; <2Mn; <1Si; <0.003C	190	490	193	40
CoCr alloys:					
CoCrWNi (F90) (ASTM, 2007a)	Co; 19-21Cr; 14–16W; 9-11Ni	310	860	210	20
CoNiCrMo (F562) (ASTM, 2007b)	Co; 33-37Ni; 19-21Cr; 9-10.5Mo	241	793	232	50
Ti and its alloys:					
Pure Ti grade 4 (F67) (ASTM, 2006)	Ti; 0.05 N; 0.1C; 0.5Fe; 0.015H; 0.4O	485	550	110	15
Ti6Al4V (F136) (ASTM, 2008)	Ti; 5.5–6.75Al; 3.5-4.5V; 0.08C; 0.2O	795	860	116	10
Degradable metals:					
Pure iron (Goodfellow, 2007)	99.8Fe	150	210	200	40
WE43 magnesium alloy (ASTM, 2001)	Mg; 3.7-4.3Y; 2.4-4.4Nd; 0.4-1Zr	150	250	44	4

**Fig. 3:**
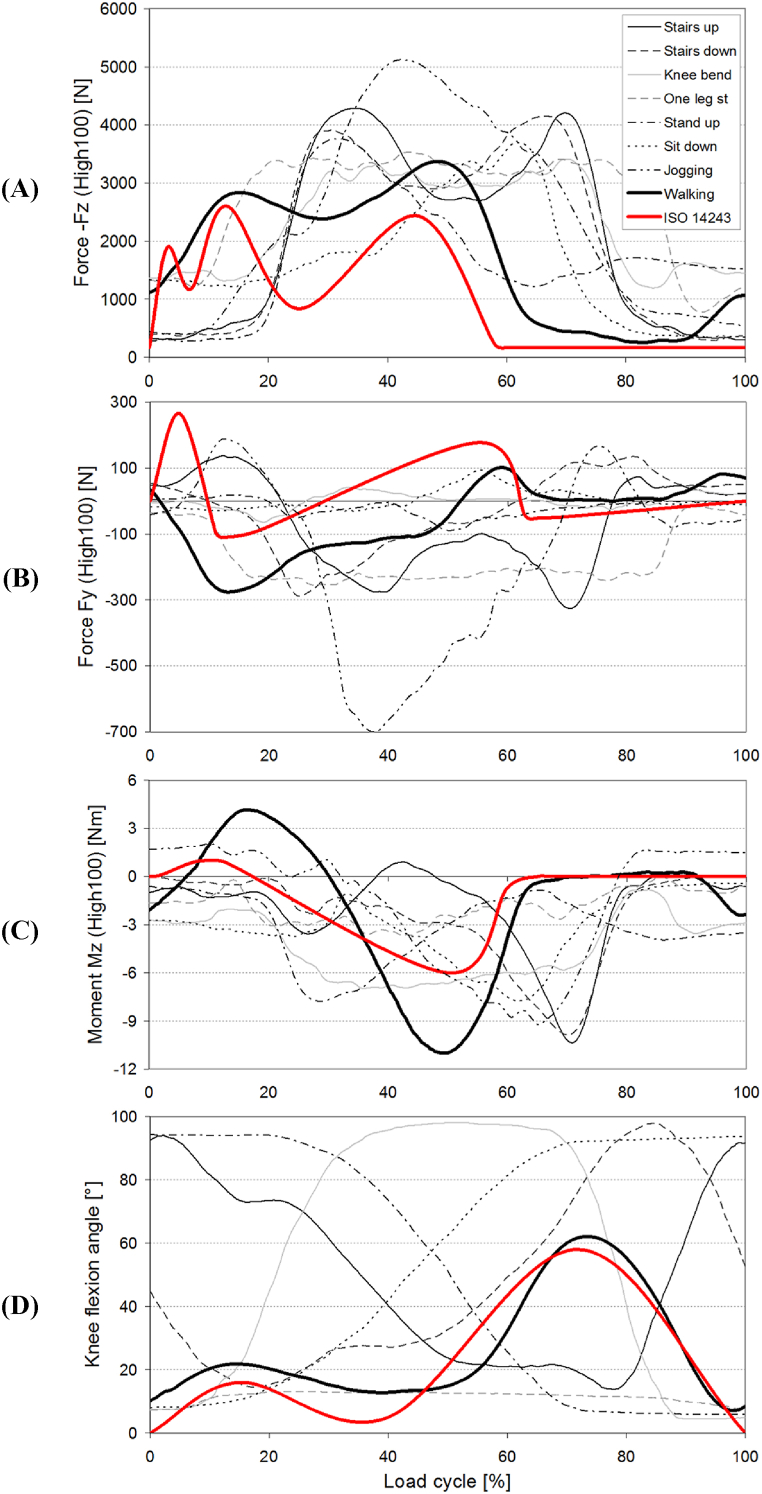
Comparison of measured load components and knee flexion angle with wear test standard; Average time courses of measured HIGH100 load components during all investigated activities and comparison with ISO 14243 wear test standard: (A) –Fz. (B) Fy. (C) Mz. (D) knee flexion angle. Extracted from Bergmann et al. [97].

As well, the loads acting in implants must be known for improving joint replacement, surgeries, physiotherapy, and numerical simulations. For this reason, Bergmann et al. delved into the intricacies of quantifying forces and torques exerted on knee joints through instrumented implants in eight subjects [97]. Their meticulous assessments highlighted that the forces during most activities, barring jogging, oscillated between 3372 and 4,218 N (Fig. 3). However, during slow jogging, a noteworthy peak force of 5,165 N was observed. Moreover, walking elicited a unique biomechanical response, generating a peak torque around the knee implant of 10.5 Nm, which, intriguingly, exceeded the torques from other activities, including jogging. These empirical observations shed light on potential discrepancies in the prevailing ISO 14243 wear test standards, as the research findings indicated force levels often surpassing the standards' guidelines. Such revelations underscore the imperative to revisit and recalibrate these standards, ensuring they robustly reflect real-world knee implant stresses, ultimately fortifying their longevity and efficacy.

**Fig. 4:**
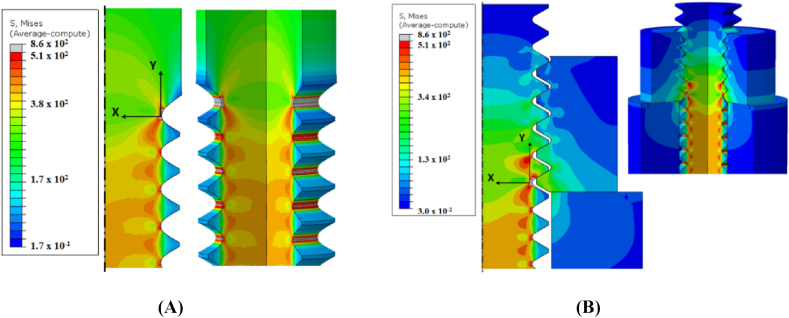
Local coordinate system to quantify stress from the root of the thread: (A) without nut interface. (B) with nut interface. Extracted from Marcelino dos Santos et al. [98].

**Fig. 5:**
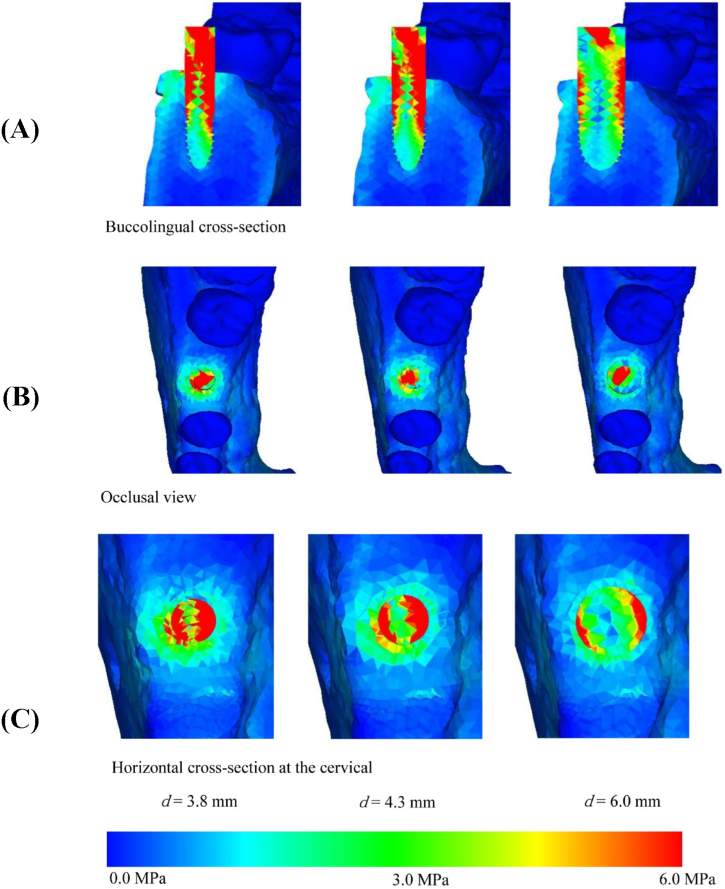
Stress distribution on the implant. (A) Buccolingual cross section [48]. (B) Occlusal view. (C) Horizontal cross-section at the cervical. Extracted from Tribst et al. [99].

Otherwise, all of the already listed implants have a combination of screws, plates, rods, hooks, and other components, but the screws are primarily responsible for holding everything together and securing it to the bones. The screw is basically a spiral inclined plane, combined with a wheel and axle that translates a rotational movement into a linear movement. The screw is fixed through friction eventually providing compression between the parts held by the threads and the nut or head of the screw. Fig. 4 illustrates a local coordinate system quantifying stress from the root of the thread without nut interface (on the left) and with nut interface (on the right) highlighting where this compression comes from. The same principle applies in implantoplasty like can be seen in Fig. 5.

## 3 Application of nanomechanical materials in medical field

Nanotechnology is enabling technology that deals with nano-meter sized objects. It is anticipated that nanotechnology will advance on a number of platforms, including materials, devices, and systems [100]. Living organisms are built of cells that typically measure less than 10 μm in diameter. However, organelles and intracellular molecules are much smaller elements than the cell and are in the sub-micron size domain. Proteins, for example, have a size of some 5 nm, which is comparable to the size of the smallest man-made nanoparticles. This simple size comparison gives an idea of how very small nanoparticle probes could be used to observe biological processes without causing too much disruption [101].

Few examples of nanomaterial applications in the biological, pharmaceutical and medical fields include: fluorescent biological dyes [102,103], drug and gene delivery [104,105], bio-detection of pathogens [38], detection of proteins [106], probing of DNA structure [40], tissue engineering [107,108], tumor destruction via heating [109], separation and purification of biological molecules and cells [109], MRI contrast enhancement [110] and phagokinetic studies [111,112].

Semiconductor nanocrystals were prepared for use as fluorescent probes in biological fluid staining and diagnostics. Many sizes of nanocrystals may therefore be excited with a single wavelength of light, emitting many colors that may be detected simultaneously. For instance, two different size CdSe–CdS core-shell nanocrystals encased in a silica shell were used to fluoresce 3T3 murine fibroblast cells***.*** The smaller nanocrystals (2-nm core) emitted green fluorescence (maximum 550 nm, 15% quantum yield), while the largest (4 nm core) emit red fluorescence (maximum 630 nm, 6% quantum yield). The surface of the nanocrystals may be tailored to interact with the biological sample either through electrostatic and hydrogen-bonding interactions or through a specific ligand-receptor interaction, such as the avidin-biotin interaction [113]. Nanocrystals coated with trimethoxysilylpropyl urea and acetate groups were found to bind with high affinity in the cell nucleus. The avidin-biotin interaction, which serves as a model system for ligand-receptor binding, was used to specifically label F-actin filaments with red nanocrystal probes. Unlike traditional multicolor dye imaging, light from a mercury lamp with a fluorescein isothiocyanate excitation filter and a single long-pass detection filter (515 nm) was used with the wide-field microscope to see both colors simultaneously. The nanocrystal-labeled samples displayed extremely minimal photobleaching over several scans, significantly less than with traditional dye molecules. The development of biological labeling nanocrystals creates new avenues for multicolor experimental research and diagnostics. Additionally, it introduces a new class of fluorescent probes for which a small organic molecule counterpart does not yet exist. Thus, these nanocrystal probes work in conjunction with current fluorophores and, in certain situations, may even outperform them [102].

Nowadays, carbon nanotubes (CNTs) have arisen in the field of nanotechnology because of their nanosize and unique features. CNTs are hollow, well-ordered carbon graphitic nanomaterials with a variety of properties. These include a high aspect ratio, a large surface area, and an ultralight weight [114]. CNTs are typically classified as single-walled (SWCNT) or multiwalled (MWCNT). SWCNTs are made up of a single cylindrical carbon layer with a diameter ranging from 0.4 to 2 nm depending on the temperature at which they were created [115]. MWCNTs are mainly formed from several cylindrical carbon layers with inner tube diameters ranging from 1 to 3 nm and outer tube diameters ranging from 2 to 100 nm [116]. The following are examples of CNT applications in medicine and biology.

### 3.1 Carbon nanotubes applications in biology and medicine

#### 3.1.1 Carbon nanotubes for cancer therapy

SWCNTs and MWCNTs have been extensively studied for their potential applications in cancer therapy. Here, we delve deeper into the nanoscale phenomena underlying their capabilities and also touch upon the limitations associated with each method.

SWCNTs have shown a unique advantage over MWCNTs in drug delivery due to their one-dimensional structure. At the nanoscale, SWCNTs provide an extremely large surface area relative to their volume. This enables them to load more therapeutic molecules, making them highly efficient carriers [115]. Furthermore, the structure of SWCNTs ensures that once they're functionalized with drugs, they present a longer circulation time in the blood compared to free drugs. This translates to prolonged and enhanced uptake by tumor cells. The enhanced permeability and retention effect refers to the phenomenon where solid tumors often have leaky blood vessels, allowing nanoscale particles to preferentially accumulate while simultaneously retaining them longer [117]. This nanoscale targeting strategy makes drug delivery more specific and effective.

However, SWCNTs are not just passive carriers. Once they have released the drug into a specific location, they undergo a clearance process. Studies indicate that they mostly get cleared through the biliary pathway and are eventually excreted in the feces, reducing the potential systemic toxicity [118].

Recent research indicates that SWCNTs can be specifically targeted. For instance, by coupling them with "Helix pomatia agglutinin" or HPA, a lectin, through carboxylic-acid modification, it's possible to target specific cancer cells like MCF-7 breast cancer cells [119]. Another study by Yang et al. has pointed out that by attaching poly(ethylene glycol) (PEG) and polyethylenimine (PEI) to SWCNTs (SWCNT-PEG-PEI), the therapeutic effects and drug delivery potential are enhanced, especially under acidic conditions commonly found in tumor microenvironments [120].

While SWCNTs excel in drug delivery, MWCNTs shine in the arena of thermal treatment. At the nanoscale, when MWCNTs are exposed to near-infrared light, they release vibrational energy. This is because they have a multilayered structure with more available electrons per particle and contain a higher percentage of metallic tubes [121]. When this energy is imparted within a tissue, it leads to localized heating. This heating is precise enough to destroy cancer cells without significantly affecting surrounding healthy tissue.

Carbon nanotubes exhibit significant potential for cancer therapeutics, but they also come with notable limitations. Concerns about the biocompatibility and potential long-term toxicity of SWCNTs arise, especially with uncertainties surrounding efficient excretion, which might lead to harmful accumulation in the body. Despite their large surface area, optimizing drug loading and release kinetics on SWCNTs remains a complex task. For MWCNTs used in thermal treatments, depth penetration of near-infrared light can be restrictive, potentially impeding the treatment of deeper tumors. Localized heating, though a strength, carries the inherent risk of inadvertently damaging neighboring healthy tissues if not meticulously controlled. Moreover, challenges related to consistency, purity, and production cost persist, complicating the scalability of functionalized carbon nanotubes for clinical use.

#### 3.1.2 Carbon nanotubes for infection therapy

Over the years, antibiotic-resistant bacteria have been responsible for hundreds of thousands of human deaths. This alarming rise in resistance against conventionally used antibiotics poses critical challenges in medical treatment [122]. Carbon nanotubes have emerged as a potential solution to this growing issue. Their antimicrobial action is attributed to unique nanoscale phenomena. For instance, CNTs' high aspect ratio and sharp edges can physically puncture bacterial cell membranes, causing the leakage of cellular contents and eventual bacterial cell death [123]. Additionally, through electrostatic interactions, positively charged functionalized CNTs can bind more strongly to the negatively charged bacterial membranes, leading to increased membrane stress and permeability [124]. Moreover, CNTs can facilitate the generation of reactive oxygen species, inducing oxidative stress in bacterial cells [125]. However, the use of CNTs as antimicrobials is not without limitations. Concerns arise regarding their potential toxicity to human cells and the environmental implications of their widespread use. Furthermore, the scalability and consistency of producing medically viable CNTs remain challenges [126]. As the demand for new antimicrobial medications increases, CNTs offer a promising but complex avenue for future research and applications [[127], [128], [129]].

#### 3.1.3 Carbon nanotubes for gene therapy

Gene therapy has emerged as a revolutionary approach to treating previously incurable diseases, encompassing cancer and numerous genetic disorders. A notable advancement in this realm is the use of CNTs for gene delivery. CNTs offer a unique nano-scale platform; their high surface area allows them to bind to various molecules, enhancing interactions with cellular components including genetic materials [130,131]. In fact, a crucial nanoscale phenomenon is the van der Waals forces which facilitate the non-covalent binding of CNTs to DNA molecules, ensuring stability and protection of the genetic material [63]. This binding aids not just in cellular uptake but also in intracellular trafficking, helping the DNA navigate the complex environment within cells [132]. Another dimension of their adaptability is the ease with which they can be functionalized to improve biocompatibility and targeting, ensuring more precise delivery [133].

However, the use of CNTs in gene therapy also comes with limitations. Potential cytotoxicity remains a concern, with some studies indicating that CNTs can induce oxidative stress or inflammatory responses in cells [134]. The heterogeneity in CNT synthesis could also lead to inconsistent results in gene delivery efficacy. Moreover, while CNTs can penetrate cells, it remains difficult to ensure that CNTs reach the genetic material in the right cell compartment (such as the nucleus). Lastly, the long-term biocompatibility and degradation profile of CNTs *in vivo* need further exploration [135].

#### 3.1.4 Carbon nanotubes for vaccines

Meng & Al. worked on the association of MWCNTs to tumor lysate protein to create a vaccine for the stimulation of immunity against a hepatocarcinoma tumor-bearing mice [136]. Experimental results showed that subcutaneous injection of carbon nanotubes considerably induced complement activation, promoted the production of inflammatory cytokines, and induced macrophage phagocytosis and activation. All of these reactions boosted the host immune system's overall activity and slowed the growth of tumor [102].

However, this therapeutic strategy does not come without challenges. There's a risk that the body might develop an immune response against the MWCNTs themselves, which could detract from the targeted tumor response or even lead to adverse side effects [137]. Moreover, long-term biocompatibility of MWCNTs remains a question, with concerns about their accumulation in organs or potential long-term toxicity [134]. The heterogeneity in MWCNT synthesis could also influence their efficiency and predictability as therapeutic agents [138].

## 4 Numerical simulation tools for medical applications

Simulation tools are increasingly used in medical applications to model the behavior of complex biological systems and to evaluate the effectiveness of different interventions and treatments. These tools use mathematical equations [139] and computational methods to simulate the behavior of biological systems, such as the cardiovascular system, respiratory system, nervous system, or musculoskeletal system. Table 4 lists different simulation tools used in medicine, their governing equations, and applications.

**Table 4:** Simulation tools used in medicine.

Simulation tool	Governing equations	Application
Finite Element Analysis (FEA)	Equations of motion and stress based on principles of continuum mechanics	Study mechanical behavior of bones, muscles, and organs [[140], [141], [142], [143], [144]].
Computational Fluid Dynamics (CFD)	Navier-Stokes equations describing motion of fluids	Study fluid dynamics in cardiovascular and respiratory systems [[145], [146], [147], [148]].
Agent-Based Modeling (ABM)	Equations based on population dynamics and cellular behavior	Study behavior of individual cells and bacteria and their interactions [[149], [150], [151], [152], [153]].
Multi-Scale Modeling (MSM)	Various equations depending on modeling approach	Study interactions between different components of biological systems [[154], [155], [156], [157], [158]].

In this context, we will focus on CFD as a simulation tool for studying the cardiovascular and respiratory systems.

## 5 Finite element simulation and computational fluid dynamics applied in human cardiovascular system

Finite element simulation and computational fluid dynamics (CFD) have revolutionized the field of medicine, enabling researchers and clinicians to gain valuable insights into complex physiological systems. These computational techniques offer powerful tools for investigating various aspects of the human body, including the cardiovascular system and respiratory system, particularly the lungs. In this discussion, we will explore the role of finite element simulation and CFD in understanding the mechanics, hemodynamics, and disease processes of the cardiovascular system and respiratory system.

The human cardiovascular system comprises a sophisticated network of blood vessels, the heart, and related structures that work together to distribute oxygenated blood to various organs and tissues. A comprehensive understanding of the mechanics and hemodynamics of this system is vital for effectively diagnosing and managing cardiovascular diseases. In recent years, the utilization of finite element simulation, combined with computational fluid dynamics (CFD), has emerged as a valuable tool for investigating and analyzing cardiovascular function and diseases [159]. Through the application of finite element modeling and CFD techniques, researchers and clinicians can obtain valuable insights into cardiovascular mechanics, blood flow dynamics, and the progression of diseases [160].

### 5.1 Coupling finite element modeling and CFD

Finite element modeling discretizes complex cardiovascular structures, such as the heart and blood vessels, into smaller elements, allowing for the accurate representation of their geometries and mechanical behavior. Each element possesses specific properties, such as material properties and boundary conditions, which are used to solve governing equations for fluid flow and structural mechanics. In the context of the cardiovascular system, finite element modeling provides a comprehensive understanding of cardiac mechanics, stress distribution, and deformation under different physiological and pathological conditions.

CFD focuses on analyzing fluid flow patterns and associated phenomena within the cardiovascular system. By solving the Navier-Stokes equations, CFD simulations offer insights into blood flow dynamics, pressure gradients, and forces acting on the vascular walls. In the context of the cardiovascular system, CFD enables the investigation of various factors influencing blood flow patterns, such as vessel geometry, vessel compliance, and cardiac function. These simulations aid in understanding hemodynamics, identifying regions of abnormal flow, and assessing the impact of cardiovascular diseases on blood flow dynamics.

The coupling of finite element modeling and CFD techniques in cardiovascular simulations allows for a comprehensive analysis of fluid-structure interactions within the cardiovascular system. This integration considers the heart and blood vessels as a fluid-structure interaction (FSI) system, where the dynamics of blood flow interact with the deformations of cardiovascular tissues. By combining finite element modeling and CFD, researchers can simulate the complex interplay between blood flow and the mechanical response of the cardiovascular system. This coupling provides a more accurate representation of cardiovascular mechanics, capturing the influence of fluid forces on the cardiac function and vascular health.

### 5.2 Advancements in cardiovascular research using finite element simulation and CFD

Finite element-based CFD simulations have greatly contributed to our understanding of cardiac function and hemodynamics. These simulations provide insights into blood flow patterns, pressure gradients, and forces acting on the cardiac chambers and valves. By quantifying flow velocities, wall shear stresses, and pressure distributions, researchers gain insights into cardiac performance, identifying regions of abnormal flow, and evaluating the impact of cardiovascular pathologies. This knowledge aids in the diagnosis and management of conditions such as valvular diseases, congenital heart defects, and cardiac remodeling.

Building upon this, Mittal et al. [161] provided a comprehensive overview of the current status and future prospects of modeling and simulating cardiac hemodynamics, with a specific focus on blood flow within the heart chambers. They presented computational modeling and simulation as a promising approach, enabled by increasingly powerful and cost-effective computing capabilities, to address the challenges posed by cardiovascular diseases. The paper emphasized the potential applications of computational modeling in enhancing diagnostic procedures, such as improving risk assessment and enabling more effective automated cardiac auscultation. Additionally, the authors identified opportunities for surgical planning in conditions like obstructive hypertrophic cardiomyopathy, highlighting procedures such as myocardial resection, leaflet plication, valve repair, prosthetic valve implantation, and patient-specific optimization of left ventricular assist devices. The authors also discussed the challenges associated with modeling cardiac flows, including the need for computational speed, efficient conversion of clinical images, appropriate valve models, and validation methods for patient-specific models. They stressed the importance of incorporating additional physics and leveraging emerging computing trends to fully realize the clinical potential of computational models in cardiac hemodynamics.

As well, Basri et al. [162] investigated the hemodynamic effects of different valve opening for 45°, 62.5°, and fully opening using a combination of magnetic resonance imaging (MRI) and CFD modeling. To identify blood behavior, the researchers assessed the hemodynamic characteristics of severed aortic stenosis in terms of pressure, velocity, and wall shear stress. The results revealed a significant drop in blood pressure at the tiny valve opening, causing blood ejection to be obstructed due to valve narrowing. As a result, the study discovered that the reduced leaflet opening decreased blood flow and increased stress on the leaflets.

Despite their role in understanding of cardiac function and hemodynamics, finite element-based CFD simulations play a crucial role in studying atherosclerosis and plaque formation within the blood vessels. These simulations capture the interaction between blood flow, vessel geometry, and plaque buildup, providing insights into flow disturbances, wall shear stresses, and regions prone to plaque deposition. By evaluating the impact of different risk factors, such as hypertension and cholesterol levels, CFD simulations aid in understanding plaque progression, assessing the vulnerability of plaques, and optimizing therapeutic strategies for preventing plaque rupture and subsequent cardiovascular events.

Recently, Pleouras et al. [163] developed and validated a multi-level plaque growth model using serial CTCA (computed tomography coronary angiography) imaging data from 94 patients. The model was designed to simulate the key mechanisms involved in the progression of atherosclerotic plaque. Through their research, they demonstrated a strong correlation between the simulated arterial geometries and the actual follow-up geometries obtained from the patients. Moreover, the authors explored the predictive capabilities of the plaque growth model in terms of lumen area reduction and plaque area increase. Their findings revealed an accuracy of 80% in predicting these disease progression parameters. In their paper, Wong et al. [164] focused on the characterization of atherosclerotic plaque using medical imaging modalities and the role of numerical simulation in predicting high-risk plaque rupture. They emphasized the significance of subintimal plaque structures, such as the fibrous cap, calcification gap, and lipid core, in determining plaque vulnerability. The authors highlighted the complex nature of calcifications within the plaque, including macrocalcifications and cellular calcifications, and their independent effects on plaque stability. They developed a model to simulate the collective effect of calcification clusters and their distance from the fibrous cap. The authors acknowledged the assumptions made in their model and the need for further adjustments based on patient-specific characteristics. Overall, their study provided insights into the role of plaque morphology and calcification structures in plaque vulnerability and offered a framework for analyzing plaque development and rupture.

Also, CFD simulations can capture the impact of altered vessel geometry, flow dynamics, and structural mechanics on the development and progression of aortic pathologies. By analyzing wall stresses, flow patterns, and the risk of rupture, these simulations aid in risk assessment, informing treatment decisions, and optimizing surgical interventions for aortic aneurysms and dissections. For instance, Soudah et al. [165] conducted a study to correlate the geometric indices of patient-specific abdominal aortic aneurysm (AAA) models with hemodynamic loads to assess the potential risk of AAA rupture. They focused on analyzing the mechanical factors that contribute to AAA failure, including maximum stress values and pressure distributions over the AAA wall. The study utilized computational fluid dynamics (CFD) to analyze the wall shear stress (WSS) distributions and flow patterns in the aneurysm sac. The findings showed that irregular flow, non-uniform WSS distribution, and high WSS values in the curvatures of the aortic vessel were associated with pathological cases. The presence of tortuosity and high saccular index affected flow fields and had significant effects on wall stress distribution. The study provided valuable insights into the relationship between geometric factors, hemodynamic loads, and the potential for thrombus formation and AAA enlargement.

**Fig. 6:**
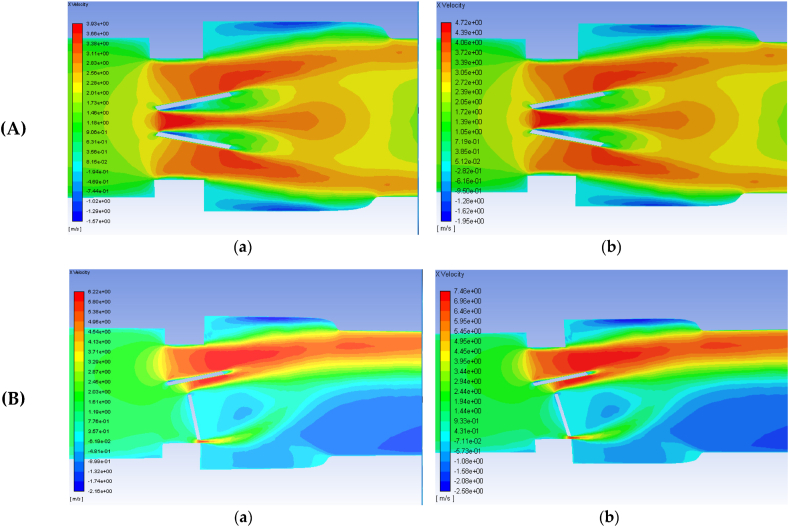
Axial velocity contours on the plane of symmetry for the: **(A)** functioning valve. **(B)** malfunctioning valve with blood and an inlet velocity of 1.25 m/s (**a**) and 1.5 m/s (**b**). Flow direction is from left to right. Extracted from James et al. [166].

As well, by simulating the behavior of the valve leaflets, fluid flow patterns, and forces acting on the valves, CFD simulations aid in diagnosing valve diseases, assessing valve function, and optimizing surgical or transcatheter interventions. For example, James et al. [166] developed a three-dimensional CFD model of a bileaflet artificial heart valve in both functioning (Fig. 6, A). and malfunctioning positions (Fig. 6, B). They compared the simulation results with existing literature to validate the computational methods used. The velocity profiles and contours of the two valves were also compared. The analysis focused on eddy intensity and distribution throughout the flow field, as well as the prediction of hemolysis using an empirical model. The results demonstrated good agreement between the functioning valve simulation and literature data, confirming the accuracy of the model. The malfunctioning valve showed a larger number of small eddies near the open leaflet, indicating increased turbulence and higher dissipation rates. Higher flowrates corresponded to increased eddy intensity and distribution, leading to greater hemolysis. However, the predicted hemolysis was found to be lower compared to previous studies, suggesting that current artificial heart valves do not cause significant hemolysis when functioning properly. The study highlights the importance of subhemolytic and sublethal damage for further valve improvement and suggests using hemolysis predictions as a comparative measure for assessing potential damage to red blood cells.

On the other hand, Tan et al. compared the aortic flow pattern before and after transcatheter aortic valve deployment in a patient-specific evaluation of a stenosed aortic valve. Using CFD simulation and an MRI data scan, the authors investigated the flow patterns of thoracic aortas in terms of velocity profile and wall shear stress. Flow patterns after implantation show a 20% reduction in jet flow at instantaneous velocity streamlines and a lower time-averaged wall shear stress [167]. In addition, Sirois et al. studied the implantation of an aortic valve on a patient-by-patient basis using CT scans and CFD simulations. The authors conducted a quantitative examination of hemodynamics in terms of blood flow patterns before and after implantation. Following valve implantation, the pressure drop was reduced to 25.27 mmHg, and the effective orifice area increased from 0.53 to 1.595 cm^2^ [168].

By simulating the impact of interventions such as stent placement, valve replacements, or bypass grafting, these simulations aid in predicting procedural outcomes, optimizing device selection, and refining interventional techniques. Moreover, CFD simulations contribute to the design and evaluation of novel cardiovascular devices, facilitating innovation and improving patient care. For example, Gao et al. evaluated the use of stent placement as an interventional technique for the treatment of coronary artery disease. The researchers analyzed blood flow before and after stent insertion and examined factors such as wall shear stress and blood velocity. The study found that the blood velocity and wall shear stress are higher before the stent is implanted, which causes a decrease in the coronary artery's maximum flow rate and an increase in wall shear stress after the procedure [169].

### 5.3 Limitation of CFD applied in human cardiovascular system

While CFD has provided numerous benefits and advanced our understanding of cardiovascular flow, it's crucial to acknowledge its limitations, especially when applied to the intricate and multifaceted nature of the human cardiovascular system.

## 6 Complex geometry and boundary conditions

The human cardiovascular system is an intricate network of vessels, each with varying diameters, bifurcations, and curvatures. Properly representing these geometries is challenging, and even small discrepancies can affect the results. Additionally, boundary conditions, such as those at vessel walls or valves, can be challenging to specify accurately [[170], [171], [172]].

## 7 Blood as a Non-Newtonian fluid

While many CFD studies treat blood as a Newtonian fluid for simplicity, it's crucial to understand that blood exhibits non-Newtonian behavior, especially under certain flow conditions. This means that its viscosity changes with the rate of shear, largely due to the presence of red blood cells [173].

## 8 Transient flow dynamics

While steady-state simulations offer computational simplicity and faster results, the cardiovascular system is inherently pulsatile due to the rhythmic nature of heartbeats. Transient or time-dependent simulations are more representative but are computationally more intensive.

## 9 Patient-specific variation

Human cardiovascular systems exhibit considerable variability across individuals. While CFD can be used for patient-specific simulations using medical imaging data, this requires much higher computational resources and is not always feasible in clinical settings [172].

## 10 Limitations of current imaging modalities

Medical imaging techniques, such as MRI or CT, used to obtain cardiovascular geometries for CFD modeling, have their own set of limitations. They might not capture the exact vessel wall details, and minor artifacts can lead to inaccuracies in CFD results [147].

## 11 Computational cost

Detailed and accurate CFD simulations, especially those considering transient dynamics, non-Newtonian flow, and intricate geometries, are computationally expensive and time-consuming. This limits their use in real-time clinical decisions.

## 12 Model validation

Validating CFD models against real-world physiological measurements is challenging due to the invasive nature of acquiring detailed in-vivo flow data. Without rigorous validation, CFD predictions might remain speculative [174].

### 12.1 Challenges and future directions

The integration of advanced imaging techniques, such as computed tomography (CT) and magnetic resonance imaging (MRI), with finite element simulation and computational fluid dynamics (CFD) holds promise for enhancing cardiovascular simulations. By incorporating imaging data into computational models, these integrated approaches enable more accurate patient-specific simulations, capturing individual anatomical and physiological variations. Continued advancements in high-performance computing (HPC) further support detailed and complex cardiovascular simulations, allowing for finer anatomical details, patient-specific variations, and real-time simulations. Moreover, the integration of machine learning algorithms and data-driven approaches with finite element simulation and CFD has the potential to advance cardiovascular research and clinical practice. By leveraging large datasets, these approaches can improve the accuracy of computational models, enhance risk prediction, optimize treatment strategies, reduce computational costs, improve simulation efficiency, and predict patient-specific outcomes.

### 12.2 Finite element simulation and computational fluid dynamics applied in human respiratory system

The respiratory system, encompassing the lungs and associated airways, plays a vital role in facilitating gas exchange and maintaining oxygenation throughout the body. Understanding the intricate mechanics of the respiratory system is crucial for diagnosing and treating respiratory diseases effectively. Finite element simulation, in conjunction with computational fluid dynamics (CFD), has emerged as a powerful tool for investigating and analyzing the behavior of respiratory systems. By employing finite element modeling and CFD techniques, researchers and clinicians gain valuable insights into respiratory mechanics, respiratory diseases, and personalized medicine.

### 12.3 Coupling finite element modeling and CFD

The coupling of finite element modeling and CFD techniques in respiratory simulations allows for a comprehensive analysis of fluid-structure interactions within the respiratory system. This integration considers the lung as a fluid-structure interaction (FSI) system, where the dynamics of fluid flow interact with the deformations of lung tissue. By combining finite element modeling and CFD, researchers can simulate the complex interplay between airflow and the mechanical response of the respiratory system. This coupling provides a more accurate representation of respiratory mechanics, capturing the influence of lung deformation on airflow patterns and vice versa.

### 12.4 Advancements in respiratory research using finite element simulation and CFD

Finite element-based CFD simulations have greatly contributed to understanding airflow distribution within the lungs and its impact on overall pulmonary function. These simulations allow for the analysis of regional ventilation, respiratory parameters, and gas exchange efficiency. By quantifying airflow velocity, pressure gradients, and ventilation-perfusion ratios, researchers gain insights into respiratory function and identify regions of abnormal ventilation. This knowledge aids in the diagnosis and management of respiratory disorders, optimizing treatment strategies for improved patient outcomes.

Tsega et al. [175] recently employed CFD simulations to examine respiratory airflow dynamics in human upper airways in response to walking and running during oral breathing. The numerical simulations were done in a realistic CT-scan airway model using ANSYS Fluent 19.0 software. In the airway model, flow patterns were examined during inspiration and expiration in response to walking and running, and flow fields were numerically assessed. Running resulted in more mixing of flow streamlines than walking due to higher turbulence incidence. When walking and running, inspiration had more skewed flows at airway curvatures than expiration. Running had a greater influence on the axial velocity distribution in human upper airways than walking. Segal et al. investigated the changes in respiratory flow patterns of four different human nasal cavities using MRI images and CFD simulations [176]. The research was carried out by numerically simulating steady-state inspiratory laminar airflow at a rate of 15 L/min and comparing the observations in terms of streamline patterns, velocities, and helicity values.

As well, CFD simulations can capture the interaction between inhaled particles and the complex lung geometry, predicting deposition patterns of pharmaceutical aerosols or environmental pollutants. Understanding particle deposition aids in optimizing drug delivery methods, ensuring targeted deposition in specific lung regions and enhancing treatment efficacy. Moreover, these simulations contribute to the evaluation of inhaler device designs, leading to the development of more efficient and patient-friendly delivery systems. For example, Dastoorian et al. [177] conducted a CFD study to investigate the influence of flow rates and cone angles on spray plume characteristics and drug particle deposition in a simulated mouth-throat airway. They validated their CFD model using experimental data. They observed that increasing the flow rate resulted in deagglomeration of larger particles and the production of finer particles. Mouth particle deposition remained relatively constant with increasing flow rates, while throat particle deposition gradually decreased due to insufficient drag force. Changing the cone angle primarily affected mouth particle deposition, with particle deposition being more dependent on the cone angle rather than the flow rate. Smaller cone angles led to the production of larger particles, particularly at the centerline, and finer particles were trapped at the 90° bend due to Brownian motion. Higher cone angles directed particles towards the inhaler nozzle and mouth cavity boundaries, increasing mouth particle deposition and reducing the efficiency of drug delivery. At a higher flow rate and an 8° cone angle, larger particles tended to move along the centerline, resulting in reduced mouth deposition.

**Fig. 7:**
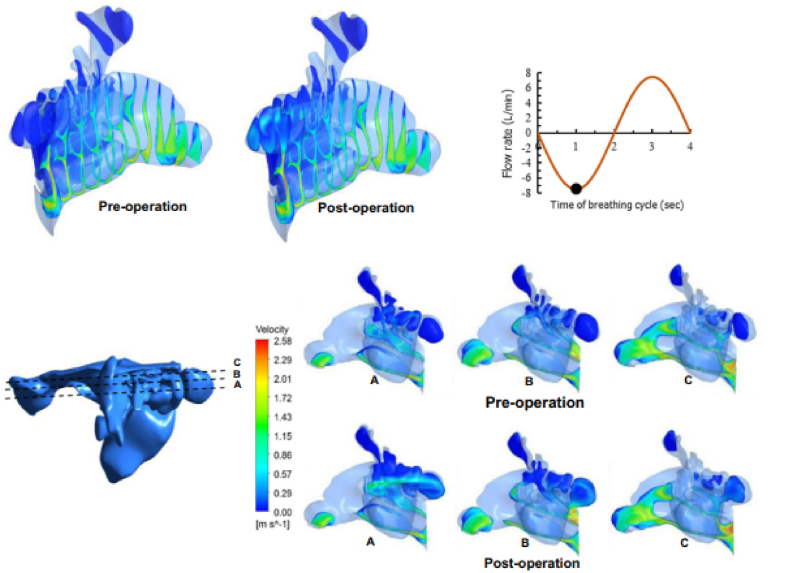
Velocity magnitude contours for various cross sections (A, B and C) at peak inhalation for a breathing intensity with peak value of 7.5 L/min. Extracted from Bahmanzadeh et al. [179].

Aside from basic airflow research on the physiological function of the nose, medication deposition is critical in the treatment of many lung diseases and allergies. Many researchers have recently become interested in using CFD to study the impact of interventions such as lung resection, bronchoplasty, or stent placement, these simulations help in predicting postoperative lung function and identifying potential complications. CFD simulations contribute to personalized treatment planning, enabling surgeons to optimize surgical techniques and parameters, leading to improved postoperative outcomes and enhanced patient care. In a recent study, Ormiskangas et al. demonstrated the use of CFD simulation to investigate the consequences of inferior turbinate surgery using CBCT images taken both prior to surgery and after one year. With larger data sets, this study demonstrated that CFD may be used to quantify patient well-being as a function of airflow characteristics from the mucosal membrane. The authors concluded that CFD may have future applications in the design and assessment of nasal surgical treatments [178]. Bahmanzadeh et al. investigated the effect of endoscopic sphenoidotomy surgery on flow patterns and microparticle deposition in the human nasal airway and sphenoid sinus. The authors demonstrated transient airflow patterns before and after surgery during a full breathing cycle with cyclic flow (Fig. 7). The transport and deposition of inhaled microparticles are assessed using a Lagrangian technique to estimate the unsteady particle that enters the nasal airway during the inhalation phase of the breathing cycle. The study discovered that sphenoidotomy improved airflow and microparticle accumulation in the sphenoid area. In the post-operative condition, 25 μm particles were shown to be able to penetrate into the sphenoid area, with the maximum deposition for 10 μm particles occurring at roughly 1.5% during resting breathing [179]**.**

### 12.5 Limitation of CFD applied in human respiratory system

By modeling airflow and particle transport, CFD provides insights into various respiratory diseases, inhaler drug delivery, and respiratory mechanics. However, as with any computational tool, there are inherent limitations to its application in the respiratory system.

## 13 Complex anatomy and structures

The respiratory system's intricate branching structure, from the trachea to the alveoli, presents significant challenges in mesh generation and spatial resolution for CFD. Accurately capturing the geometries of such a bifurcating system is non-trivial [180].

## 14 Breathing dynamics

The respiratory cycle involves both inhalation and exhalation, with varying flow rates and patterns. While some studies focus on steady-state inhalation, human respiration is inherently transient, which affects airflow dynamics.

## 15 Multiscale modeling challenges

The respiratory system functions at multiple scales: from airflow in the large bronchi to oxygen-carbon dioxide exchange at the alveolar level. Capturing all these scales in one simulation is currently beyond our computational capabilities [156].

## 16 Soft tissue dynamics

Lung tissue, the trachea, and the bronchi are flexible structures. Their shapes change during breathing, altering the airflow patterns. Modeling this interaction between airflow and tissue deformation (fluid-structure interaction) accurately is computationally intensive.

## 17 Heterogeneity in lung tissue

The lung is not a uniform organ. Diseases such as chronic obstructive pulmonary disease (COPD) or asthma can cause localized obstructions or tissue alterations, leading to heterogeneity in airflow. Representing these variations in a CFD model can be challenging [181].

## 18 Model validation and in vivo measurements

Verifying and validating CFD models against real-life physiological data is imperative. Acquiring detailed in-vivo flow measurements in the respiratory system, particularly deeper in the lungs, is invasive and difficult [182].

## 19 Particle transport and deposition

For drug delivery or pollutant studies, predicting particle deposition is essential. The behavior of particles, influenced by factors like size, shape, and hygroscopic growth, makes these simulations challenging [183].

### 19.1 Challenges and future directions

Continued advances in computational power will enable more detailed and comprehensive simulations of the respiratory system, including high-fidelity simulations considering finer lung structures, multiscale modeling, and longer simulation times. This increased computational power will facilitate real-time simulations, allowing clinicians to make informed decisions during procedures and interventions. Moreover, the integration of multiscale modeling with imaging techniques such as computed tomography (CT) and magnetic resonance imaging (MRI) will enhance the accuracy and fidelity of finite element-based CFD simulations. By combining imaging data with computational models, patient-specific simulations can capture individual anatomical and physiological variations, further enhancing personalized diagnostics, treatment planning, and therapy optimization. Additionally, the application of data-driven approaches combined with machine learning algorithms can leverage large datasets to improve the accuracy of computational models, enhance understanding of respiratory function and disease progression, and aid in clinical decision-making. Machine learning techniques can also optimize simulations, reduce computational costs, and improve the prediction of patient-specific outcomes.

## 20 Conclusion

In conclusion, our comprehensive exploration of the intersection of mechanical engineering, biology, and medicine has revealed the transformative influence of mechanical principles and innovations on these multifaceted disciplines. We began our ongoing quest by emphasizing the pivotal role of advanced biomaterials, specifically polymers and composites, in reshaping medical applications. While celebrating their versatility and tailored mechanical properties, we acknowledged the challenges related to long-term biocompatibility and immune responses, highlighting the potential of polymers like PLA, PLGA, and PHA in various medical domains.

Moving forward, our investigation into biomechanics elucidated the crucial role of mechanical implants, notably in orthopedics, spinal interventions, craniomaxillofacial procedures, and orthopedic trauma interventions. These interventions underscored the profound impact of engineering on restoring function and improving the quality of life for patients. Yet, the significance of ongoing monitoring and post-surgical care cannot be overstated.

Exploring nanomechanical materials in the medical realm, particularly carbon nanotubes (CNTs), unveiled their immense potential in diverse areas, including cancer therapy, infection control, gene therapies, and vaccine development. While SWCNTs showcased prowess in drug delivery, MWCNTs demonstrated thermal treatment capabilities. Nevertheless, biocompatibility, toxicity concerns, and scalability challenges must be addressed to facilitate their clinical adoption.

Our final round into the realm of computational fluid dynamics (CFD) illuminated its indispensable role in comprehending complex physiological processes, disease mechanisms, and treatment strategies within the human cardiovascular and respiratory systems. Recent advancements in CFD techniques have propelled medical science into uncharted territories, albeit with challenges such as patient-specific modeling and computational demands. Nevertheless, the future holds promise for CFD in personalized medicine, drug development, and ongoing medical advancements.

In brief, our exploration underscores the enduring influence of mechanical engineering on healthcare and medical science. As we navigate the ever-evolving landscape of interdisciplinary collaboration, these insights serve as a testament to the dynamic synergy between mechanical principles, biology, and medicine, propelling us toward a future enriched by innovation, discovery, and enhanced human health and well-being.

## Funding

This research received no external funding.

## CRediT authorship contribution statement

**Eddie Gazo Hanna:** Writing – review & editing, Writing – original draft, Validation, Supervision, Software, Project administration, Conceptualization. **Khaled Younes:** Writing – review & editing, Writing – original draft, Resources. **Rabih Roufayel:** Writing – review & editing, Writing – original draft, Resources. **Mickael Khazaal:** Writing – original draft, Resources. **Ziad Fajloun:** Writing – review & editing, Writing – original draft, Validation, Supervision, Project administration, Conceptualization.

## Declaration of competing interest

The authors declare that they have no known competing financial interests or personal relationships that could have appeared to influence the work reported in this paper.
